# Inflammation-oriented dietary pattern and menopause timing in postmenopausal women: evidence from the Health and Retirement Study

**DOI:** 10.3389/fnut.2026.1905625

**Published:** 2026-08-12

**Authors:** Yufan Liang, Zhuoyan Huang, Zixia Li, Hanrong Li, Junxin Lu, Kexin Zhong, Wenjing Shi, Yuhua Ou

**Affiliations:** 1 Department of Gynecology, The Second Affiliated Hospital of Guangzhou Medical University, Guangzhou, China; 2 The Second Clinical Medical School, Guangzhou Medical University, Guangzhou, China; 3 The Third Clinical Medical School, Guangzhou Medical University, Guangzhou, China

**Keywords:** inflammation-oriented dietary pattern, inflammatory biomarkers, menopause timing, postmenopausal women, principal component analysis, reduced rank regression

## Abstract

**Objective:**

To develop an inflammation-oriented dietary pattern among postmenopausal women using data from the Health and Retirement Study, examine its association with inflammatory biomarkers, and secondarily explore menopause timing-related differences in the dietary score.

**Methods:**

This study included 1,426 postmenopausal women. Natural dietary patterns were identified using principal component analysis based on intake data for 40 food groups from the 2013 Health Care and Nutrition Survey; Subsequently, using log-transformed C-reactive protein, interleukin-6, and soluble tumor necrosis factor receptor 1 as response variables, we employed reduced-rank regression to extract inflammation-oriented dietary patterns and construct standardized scores. Multivariate linear regression was used to analyze the association between dietary scores and inflammatory markers, and to further explore the contribution of menopausal status and food groups.

**Results:**

Principal component analysis identified three natural dietary patterns. The reduced-rank regression inflammation-oriented dietary pattern was characterized by higher intakes of processed meat, sugar-sweetened beverages, and refined grains, along with lower intakes of nuts, fish and seafood, fruits, vegetables, and whole grains. Higher reduced-rank regression inflammation-oriented dietary scores were positively correlated with log C-reactive protein, log interleukin-6, and log soluble tumor necrosis factor receptor 1 levels. Exploratory analyses showed that women with premature or early menopause had higher inflammation-oriented dietary scores, accompanied by higher log IL-6 levels; however, the association between the timing of menopause and inflammatory markers was inconsistent, and it did not significantly modify the relationship between dietary scores and inflammatory markers.

**Conclusion:**

Among postmenopausal women in the HRS, the reduced-rank regression inflammation-oriented dietary score was associated with multiple inflammatory markers, primarily reflecting a dietary pattern characterized by higher intake of processed/refined foods and lower intake of plant-based foods, fish, and whole grains. Women who experienced earlier menopause may have higher reduced-rank regression inflammation-oriented dietary scores; however, these findings require further validation through longitudinal studies and in external cohorts.

## Introduction

The postmenopausal period represents a unique phase in the health management of middle-aged and older women. With the decline in ovarian function and falling estrogen levels, the perimenopausal and postmenopausal periods are often accompanied by changes in lipid metabolism, body fat distribution, vascular function, and the state of immune inflammation (1–4); these changes may collectively influence metabolism and overall health in middle-aged and older women. Among these biological changes, chronic low-grade inflammation is an objectively measurable phenotype that warrants attention. Markers such as C-reactive protein (CRP), interleukin-6 (IL-6), and soluble tumor necrosis factor receptors are commonly used to assess systemic inflammatory status (5, 6). Previous studies suggest that levels of certain inflammatory markers may change during the menopausal transition and the aging process in women (7, 8); simultaneously, elevated levels of inflammatory markers are associated with age-related health outcomes such as metabolic abnormalities, insulin resistance, and the risk of vascular events (9, 10). Therefore, focusing on inflammatory phenotypes in postmenopausal women and identifying modifiable factors associated with inflammation levels can help further understand the nutritional and health risk profiles of this population.

Among modifiable factors, diet is a lifestyle factor that has received considerable attention in studies of inflammatory status in postmenopausal women. A study from the Women’s Health Initiative (WHI) showed that, in postmenopausal women, higher Dietary Inflammatory Index (DII) scores were associated with higher levels of IL-6, tumor necrosis factor-*α* receptor 2, and composite inflammatory marker scores (11). Another study of a multiethnic cohort of postmenopausal women in the United States also observed that scores on the Empirical Dietary Inflammatory Pattern (EDIP) were associated with various circulating inflammatory markers (12). Furthermore, a small-sample study of postmenopausal women in New Zealand showed that the intake of foods such as fruits and vegetables was negatively correlated with multiple inflammatory markers, while dietary patterns characterized by fast-food consumption were associated with higher CRP levels (13). These studies suggest that diet may be a key clue to understanding inflammatory differences in postmenopausal women.

Health and Retirement Study (HRS) and the 2013 Health Care and Nutrition Study (HCNS) have been used for nutritional epidemiological research among middle-aged and older adults in the United States. Previous studies based on HRS-HCNS data have found that higher scores on the Healthy Eating Index-2015 are associated with a lower risk of functional limitations, depression, and poor self-rated health (14); A combined analysis of the 2013 HCNS and the 2016 Venous Blood Study (VBS) also suggests that poorer overall dietary quality is associated with higher composite biological risk (15). Recent studies have further linked the Plant-Based Diet Index and the Planetary Health Diet Index to outcomes such as multimorbidity, mortality, and cardiovascular risk (16, 17). These studies indicate that data from the HRS-HCNS and its associated biomarkers can be used to assess the relationship between dietary composition and health outcomes in middle-aged and older adults. However, existing HRS nutritional studies have primarily focused on overall dietary quality or predefined dietary indices, with few directly incorporating inflammatory biomarkers to derive inflammation-oriented dietary patterns in postmenopausal women. Furthermore, while the timing of menopause may be associated with metabolic and inflammatory status in postmenopausal women (18), there remains insufficient evidence to determine whether it is associated with differences in inflammation-related dietary patterns or whether it influences the relationship between dietary patterns and inflammatory biomarkers.

Dietary pattern analysis provides a methodological foundation for further exploring the aforementioned issues. Principal component analysis (PCA) can be used to identify naturally occurring major dietary structures within a population, but its extraction process does not directly incorporate biological information such as inflammation (19). In contrast, reduced rank regression (RRR) can simultaneously incorporate food groups and predefined biological response variables to extract food combinations that explain the variation in these response variables (20). Therefore, in dietary-inflammation research, patterns derived from RRR more directly point to the inflammatory phenotypes of interest. Combining PCA with RRR helps to simultaneously describe natural dietary structures within a population and further identify dietary dimensions that are more closely associated with inflammatory biomarkers.

Based on this, the present study utilized data from the HRS to construct and characterize inflammation-promoting dietary patterns among postmenopausal women. We first used PCA to identify naturally occurring dietary patterns in the population; subsequently, using log-transformed CRP, IL-6, and soluble tumor necrosis factor receptor 1 (sTNFR-1) as response variables, we employed RRR to extract inflammation-oriented dietary patterns and construct corresponding dietary scores. Building on this, the study further evaluated the relationship between this score and levels of inflammatory markers, and explored whether there were differences in the inflammation-oriented dietary score and its food group composition between women with premature or early menopause and those with normal menopausal age. This study aims to provide population-based evidence for understanding the relationship between dietary patterns, menopausal timing, and inflammatory phenotypes in postmenopausal women, and to serve as a reference for future research on inflammation-related dietary patterns.

## Methods

### Study population

This study is based on the HRS. The HRS is a nationally representative longitudinal study of middle-aged and older adults in the United States. Launched in 1992, the core survey is conducted every two years, and new cohorts are regularly added to maintain its representativeness of the U. S. aging population. In addition to the core questionnaire, the HRS collects supplementary data on biomarkers, genetics, and psychosocial factors, and can be linked to certain administrative records.

This study utilized dietary data from the 2013 HCNS and inflammatory marker data from the 2016 VBS. The 2016 VBS was selected as the source of inflammatory markers primarily because it provided the inflammation-related indicators required for the RRR analysis in this study—including CRP, IL-6, and sTNFR-1—within the same venous blood sampling framework. Although the HRS also collected some DBS biomarkers in survey waves closer in time to the HCNS, its combination of markers, sample sources, and testing methods was not entirely consistent with the 2016 VBS and could not provide the same set of inflammatory biomarkers from a single venipuncture required for this study. Therefore, this study conducted an association analysis using dietary data from the 2013 HCNS and inflammatory biomarkers from the 2016 VBS. The study population was restricted to women who completed the HCNS and had valid information on age at menopause, with further exclusion of those missing any of the following markers: CRP, IL-6, or sTNFR-1. A total of 1,426 postmenopausal women were ultimately included in the analysis; the specific selection process is shown in Supplementary Figure S1.

### Dietary assessment

Dietary data were obtained from the 2013 HRS HCNS. The HCNS was conducted from November 2013 to May 2014 to collect information on health care, food purchases, food intake, and nutrition from a subset of participants in the HRS follow-up sample. This study utilized data from the Food Frequency Questionnaire (FFQ) in the HCNS Food and Nutrition Module (Section C). This FFQ was developed based on the Harvard semi-quantitative FFQ and was used to assess the usual frequency and amount of consumption of various food items by participants over the past year (21).

In this study, based on the estimated daily intake of each food item in the FFQ, the original food items were grouped into 40 predefined food groups according to nutritional composition, food sources, and processing characteristics, and these groups were used for PCA and RRR analyses. Total energy intake was derived from the Nutrient Totals data released alongside the HCNS and was included as a covariate in subsequent regression models.

### Inflammatory biomarkers

The data on inflammatory biomarkers were derived from the 2016 HRS VBS. The VBS measured multiple blood biomarkers by collecting peripheral venous blood samples; all laboratory testing was conducted by the University of Minnesota Advanced Research and Diagnostic Laboratory. This study selected high-sensitivity C-reactive protein (hsCRP), IL-6, and sTNFR-1 as inflammation-related markers. To maintain consistency with the tables and results presented throughout the text, hsCRP will be referred to as CRP in the following text. CRP was measured using a latex particle-enhanced immunoturbidimetric assay on the Roche COBAS 6000 platform; IL-6 and sTNFR-1 were measured using the Simple Plex assay on the ProteinSimple ELLA platform. Since the original distributions of all three markers were right-skewed, natural logarithmic transformation was performed before analysis, and log CRP, log IL-6, and log sTNFR-1 were used for subsequent statistical analysis.

### Menopausal timing

Information on menopause was derived from the HRS variables related to female reproductive health. This study used the menopause stage variables (r9menope, r10menope, r11menope) and age at last menstrual period variables (r9lstmnspd, r10lstmnspd, r11lstmnspd), as well as the corresponding source indicator variables for age at last menstrual period (r9flstmnspd, r10flstmnspd, r11flstmnspd) to construct postmenopausal status and age at menopause.

Respondents were classified as postmenopausal if they reported being postmenopausal in any wave or had a valid record of age at last menstrual period. Age at menopause was defined as the age at last menstrual period; when constructing age at menopause, this study prioritized directly reported age at last menstrual period; when direct reports were unavailable, valid approximate or synthesized age values generated by the HRS based on available respondent responses were used. If multiple valid records existed across Waves 9–11, the earliest valid value was used in chronological order of the survey. Based on age at menopause, participants were further divided into two groups: premature or early menopause (<45 years) and normal menopausal age (≥45 years).

Regarding non-natural menopause, for HRS respondents who were not asked about their age at last menstrual period due to hysterectomy, the age at last menstrual period variable is typically recorded as a special missing value. Additionally, since the Harmonized HRS does not provide a standardized variable that can reliably distinguish between bilateral oophorectomy and medically induced menopause, this study was unable to further differentiate between these conditions; these limitations are discussed in the Limitations section.

### Covariates

Covariates included age, age at menopause, race, educational attainment, marital status, total energy intake, current smoking, current alcohol consumption, hypertension, diabetes, cancer, cardiovascular disease, and body mass index (BMI) classification. Race was categorized as White/Caucasian, Black/African American, and Other; educational attainment was categorized as less than high school/General Educational Development (GED), high school graduate, and some college or above; marital status was categorized as married/partnered, separated/divorced, widowed, and never married. Current smoking and current alcohol consumption were both included in the analysis as yes/no variables. Hypertension, diabetes, cancer, and cardiovascular disease were determined based on self-reported physician diagnoses, with cardiovascular disease defined as a history of heart disease and/or stroke. BMI was classified according to standard categories as underweight (<18.5 kg/m^2^), normal weight (18.5–24.9 kg/m^2^), overweight (25.0–29.9 kg/m^2^), and obese (≥30.0 kg/m^2^).

### Statistical analysis

Continuous variables are expressed as means and standard deviations, and categorical variables are expressed as counts and percentages. Study participants were grouped according to tertiles of the standardized RRR-oriented inflammatory dietary score, and differences between groups were compared using the Kruskal–Wallis test or the Pearson χ^2^ test. Missing values for covariates were handled using chained equation multiple imputation in this study.

PCA was performed based on 40 food groups, and data suitability was assessed using the Kaiser–Meyer–Olkin (KMO) test and Bartlett’s sphericity test (Supplementary Table S1). Factor retention was determined by comprehensively considering eigenvalues, scree plots, model parsimony, and nutritional interpretability (Supplementary Table S2; Supplementary Figure S2). The final retained factors were subjected to Varimax rotation, and food groups with absolute rotation loadings ≥0.25 were used for dietary pattern naming and interpretation. Subsequently, RRR analysis was performed using the 40 food groups as predictors and log CRP, log IL-6, and log sTNFR-1 as response variables. The first RRR-oriented pattern was retained to construct an inflammatory dietary score. The score was obtained by summing the standardized food group intakes weighted by their corresponding RRR loadings, and was further standardized to have a mean of 0 and a standard deviation of 1; a higher score indicates greater adherence to the inflammatory dietary pattern. PCA results were used to describe the natural dietary structure and to assess its overlap with the RRR-oriented inflammatory dietary pattern.

Multivariate linear regression models were used to assess the associations between the inflammatory dietary score and log CRP, log IL-6, and log sTNFR-1. The dietary score was included in the models as both a continuous variable and in tertiles; results are presented as *β* values and 95% confidence intervals. Trend tests were performed by including the median dietary score for each tertile in the models. Model 1 was adjusted for age and total energy intake; Model 2 was further adjusted for educational level, race, marital status, current smoking, current alcohol consumption, and age at menopause; Model 3 was further adjusted for hypertension, diabetes, cancer, cardiovascular disease, and BMI classification.

Because age at menopause was retrospectively reported and dietary intake was assessed after menopause, analyses involving menopause timing were considered exploratory. We compared the RRR-oriented inflammatory dietary score between women with premature or early menopause (<45 years) and those with normal menopausal age (≥45 years), assessed the direct association between menopausal timing and inflammatory markers, and included an interaction term between dietary score and menopausal timing in the model; stratified analyses were also conducted by menopausal timing. For food groups contributing to higher dietary scores among women with earlier menopause, a further food group contribution decomposition was performed to describe the relative contribution of each food group to the differences in composite scores. All analyses were conducted using R 4.5.1, and a two-sided *p* < 0.05 was considered statistically significant.

## Results

### Participant characteristics by inflammatory dietary score tertiles

The final analysis included a total of 1,426 postmenopausal women. After grouping participants into tertiles based on the standardized RRR-oriented inflammatory dietary score, the baseline characteristics of each group are shown in Table 1. Compared with the T1 group, the T3 group had lower educational attainment, a lower proportion of White/Caucasian women, and a higher proportion of Black/African American women. Regarding marital status, the T3 group had a lower proportion of married or partnered women and a higher proportion of widows. The T3 group had higher rates of obesity, current smoking, hypertension, diabetes, and cardiovascular disease, while the rates of current alcohol consumption and total energy intake were lower. The mean age at menopause was also slightly lower in the T3 group than in the T1 group. Except for cancer, most baseline characteristics showed statistically significant differences across the different score tertiles (*p* < 0.05).

**Table 1:** Baseline characteristics of participants by tertiles of the RRR-oriented inflammatory dietary score.

Characteristic	Overall *N* = 1,426	T1 (lowest) *N* = 476	T2 N = 475	T3 (highest) *N* = 475	*p* value
RRR-oriented inflammatory dietary score	0.00 (1.00)	−1.00 (1.03)	0.15 (0.20)	0.85 (0.38)	<0.001
Age, years	66.48 (9.31)	65.21 (9.09)	67.38 (9.18)	66.87 (9.54)	<0.001
Education level					<0.001
Less than high school/GED	297 (20.8%)	84 (17.6%)	77 (16.2%)	136 (28.6%)	
High school graduate	405 (28.4%)	100 (21.0%)	138 (29.1%)	167 (35.2%)	
Some college or above	724 (50.8%)	292 (61.3%)	260 (54.7%)	172 (36.2%)	
Race					0.004
White/Caucasian	1,111 (77.9%)	379 (79.6%)	389 (81.9%)	343 (72.2%)	
Black/African American	207 (14.5%)	59 (12.4%)	58 (12.2%)	90 (18.9%)	
Other	108 (7.6%)	38 (8.0%)	28 (5.9%)	42 (8.8%)	
Marital status					0.003
Married/partnered	868 (60.9%)	312 (65.5%)	293 (61.7%)	263 (55.4%)	
Separated/divorced	200 (14.0%)	68 (14.3%)	72 (15.2%)	60 (12.6%)	
Widowed	293 (20.5%)	80 (16.8%)	89 (18.7%)	124 (26.1%)	
Never married	65 (4.6%)	16 (3.4%)	21 (4.4%)	28 (5.9%)	
BMI category					0.007
Underweight	18 (1.3%)	9 (1.9%)	4 (0.8%)	5 (1.1%)	
Normal	318 (22.3%)	133 (27.9%)	94 (19.8%)	91 (19.2%)	
Overweight	418 (29.3%)	137 (28.8%)	141 (29.7%)	140 (29.5%)	
Obese	672 (47.1%)	197 (41.4%)	236 (49.7%)	239 (50.3%)	
Total energy intake, kcal/day	1,824.48 (1,146.51)	2,257.55 (1,471.38)	1,653.71 (769.05)	1,561.26 (950.68)	<0.001
Age at menopause, years	50.20 (5.18)	50.72 (5.00)	50.19 (5.08)	49.67 (5.40)	0.004
Current smoking	131 (9.2%)	23 (4.8%)	40 (8.4%)	68 (14.3%)	<0.001
Current drinking	505 (35.4%)	212 (44.5%)	178 (37.5%)	115 (24.2%)	<0.001
Hypertension	768 (53.9%)	226 (47.5%)	253 (53.3%)	289 (60.8%)	<0.001
Diabetes	273 (19.1%)	73 (15.3%)	97 (20.4%)	103 (21.7%)	0.031
Cancer	184 (12.9%)	58 (12.2%)	71 (14.9%)	55 (11.6%)	0.256
Cardiovascular disease	256 (18.0%)	63 (13.2%)	84 (17.7%)	109 (22.9%)	<0.001

Values are presented as mean (standard deviation) or *n* (%). Tertiles were defined according to the standardized RRR-oriented inflammatory dietary score. *p* values were calculated using Kruskal–Wallis tests for continuous variables and Pearson chi-square tests for categorical variables. BMI, body mass index; GED, General Educational Development; RRR, reduced rank regression.

### PCA-oriented dietary patterns

**Figure 1:**
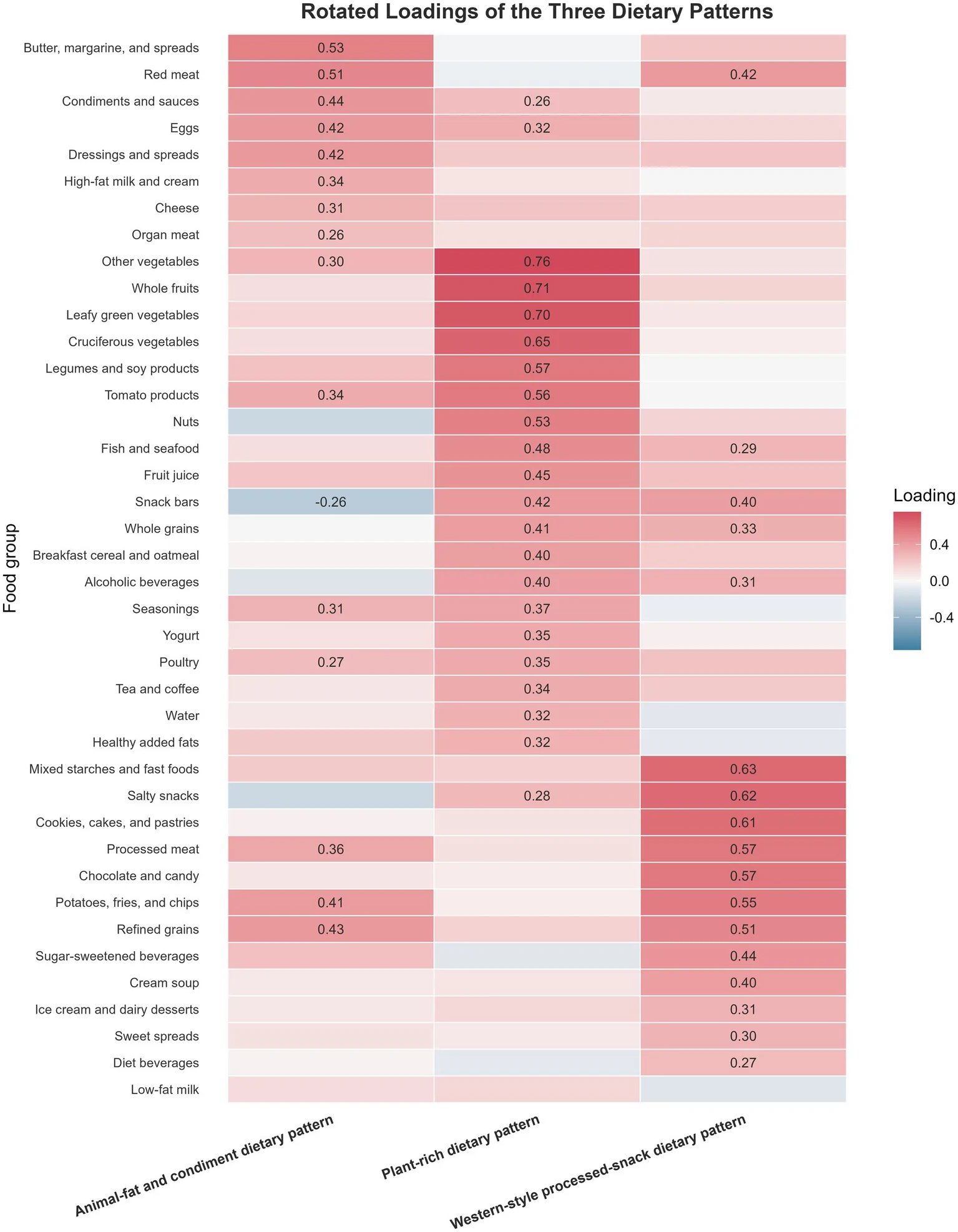
Heatmap graphic displaying rotated loadings of three dietary patterns—animal-fat and condiment, plant-rich, and western-style processed-snack—for various food groups, with color scale indicating loading strength from blue (negative) to red (positive). PCA, principal component analysis.

Following PCA analysis of 40 food groups, a three-factor solution was ultimately retained. As shown in Figure 1, Supplementary Table S3, and Supplementary Table S4, the three PCA-oriented dietary patterns collectively explained 30.17% of the total variance in food group intake. This proportion of explained variance is generally consistent with common findings from PCA analyses in dietary pattern studies; however, it also suggests that dietary differences among a significant proportion of individuals remain not fully captured by these three patterns. Therefore, the PCA results are primarily used to describe the major, nutritionally meaningful natural dietary structures observed in the study sample. The first pattern explained 13.07% of the variance and was named the “plant-rich dietary pattern.” Its main characteristics were high intakes of whole fruits, other vegetables, leafy green vegetables, cruciferous vegetables, legumes and soy products, nuts, fish and seafood, and whole grains. The second pattern explained 10.23% of the variance and was named the “Western-style processed-snack dietary pattern.” Its main characteristics were higher intakes of mixed starches and fast foods, salty snacks, cookies, cakes, and pastries, processed meat, refined grains, and sugar-sweetened beverages. The third pattern explains 6.88% of the variance and is named the “animal-fat and condiment dietary pattern.” Its main characteristics include higher intakes of butter, margarine, and spreads; red meat; condiments and sauces; eggs; dressings and spreads; high-fat milk and cream; and cheese.

### RRR-oriented inflammatory dietary pattern and overlap with PCA-oriented patterns

After conducting RRR analysis using log-transformed CRP, IL-6, and sTNFR-1 as response variables, the first RRR-oriented pattern was retained to construct an inflammatory dietary score. As shown in Supplementary Table S5, the food groups with positive loadings in this pattern primarily included processed meat, sugar-sweetened beverages, and refined grains; those with negative loadings primarily included nuts, fish and seafood, alcoholic beverages, cruciferous vegetables, whole fruits, leafy green vegetables, other vegetables, and whole grains. In other words, a higher inflammatory dietary score reflects higher intake of processed meat, sugar-sweetened beverages, and refined grains, as well as lower intake of nuts, fish and seafood, fruits, vegetables, and whole grains.

Subsequently, we compared the RRR-oriented inflammatory dietary pattern with three PCA-oriented dietary patterns. As shown in Supplementary Table S6, Supplementary Table S7, and Supplementary Figure S3, this pattern overlapped most with the plant-rich dietary pattern but in the exact opposite direction, with a weighted absolute overlap of 1.428, a signed alignment of −1.428, and all eight overlapping food groups exhibiting opposite directions. This pattern showed moderate overlap with the Western-style processed-snack dietary pattern, with a weighted absolute overlap of 0.719. Specifically, processed meat, refined grains, and sugar-sweetened beverages overlapped in the same direction, while fish and seafood, alcoholic beverages, and whole grains overlapped in the opposite direction. In contrast, the overlap between this pattern and the animal-fat and condiment dietary pattern was weaker, with a weighted absolute overlap of 0.303. Additionally, descriptive correlation analysis showed that the RRR-oriented inflammatory dietary score was positively correlated with log CRP, log IL-6, and log sTNFR-1 (Supplementary Table S8).

### Associations between the inflammatory dietary score and inflammatory biomarkers

Among the 1,426 postmenopausal women included in the main regression models, a higher RRR-oriented inflammatory dietary score was associated with higher levels of inflammatory markers. As shown in Table 2, in the analysis of continuous variables, the fully adjusted Model 3 indicated that a 1-standard-deviation increase in the RRR-oriented inflammatory dietary score was associated with higher log CRP [*β* = 0.075, 95% confidence interval (CI) (0.016, 0.133), *p* = 0.013], log IL-6 [*β* = 0.132, 95% CI (0.083, 0.180), *p* < 0.001], and log sTNFR-1 [*β* = 0.043, 95% CI (0.022, 0.065), *p* < 0.001].

**Table 2:** Associations of the RRR-oriented inflammatory dietary score with inflammatory biomarkers.

Outcome	Analysis	Model/contrast	β (95% CI)	*p* value	P for trend
log CRP	Continuous score	Model 1	0.144 (0.084, 0.204)	<0.001	
log CRP	Continuous score	Model 2	0.110 (0.048, 0.172)	<0.001	
log CRP	Continuous score	Model 3	0.075 (0.016, 0.133)	0.013	
log IL-6	Continuous score	Model 1	0.184 (0.136, 0.232)	<0.001	
log IL-6	Continuous score	Model 2	0.151 (0.101, 0.201)	<0.001	
log IL-6	Continuous score	Model 3	0.132 (0.083, 0.180)	<0.001	
log sTNFR-1	Continuous score	Model 1	0.071 (0.049, 0.093)	<0.001	
log sTNFR-1	Continuous score	Model 2	0.055 (0.032, 0.077)	<0.001	
log sTNFR-1	Continuous score	Model 3	0.043 (0.022, 0.065)	<0.001	
log CRP	Tertile score	T2 vs. T1	0.102 (−0.022, 0.225)	0.106	<0.001
log CRP	Tertile score	T3 vs. T1	0.242 (0.112, 0.371)	<0.001	
log IL-6	Tertile score	T2 vs. T1	0.111 (0.008, 0.214)	0.035	<0.001
log IL-6	Tertile score	T3 vs. T1	0.232 (0.125, 0.340)	<0.001	
log sTNFR-1	Tertile score	T2 vs. T1	0.063 (0.018, 0.108)	0.006	<0.001
log sTNFR-1	Tertile score	T3 vs. T1	0.103 (0.056, 0.151)	<0.001	

All models included 1,426 postmenopausal women. β coefficients represent differences in log-transformed inflammatory biomarkers. Continuous score analyses estimate the association per 1-standard-deviation higher RRR-oriented inflammatory dietary score. Tertile analyses used T1 as the reference group and were adjusted as in Model 3. Model 1 adjusted for age and total energy intake. Model 2 additionally adjusted for education level, race, marital status, current smoking, current drinking, and age at menopause. Model 3 additionally adjusted for hypertension, diabetes, cancer, cardiovascular disease, and BMI category. P for trend was estimated using the median dietary score within each tertile. BMI, body mass index; CI, confidence interval; CRP, C-reactive protein; IL-6, interleukin-6; RRR, reduced rank regression; sTNFR-1, soluble tumor necrosis factor receptor 1.

The results of the tertile analysis were generally consistent with those of the continuous variable analysis. Using the T1 group as a reference, the log CRP in women in the T3 group [*β* = 0.242, 95% CI (0.112, 0.371), *p* < 0.001], log IL-6 [*β* = 0.232, 95% CI (0.125, 0.340), *p* < 0.001], and log sTNFR-1 [*β* = 0.103, 95% CI (0.056, 0.151), *p* < 0.001] were all higher, and all three inflammatory markers showed a significant upward trend (all P for trend < 0.001). Supplementary Figure S4 further demonstrates that as the RRR-oriented inflammatory dietary score increased across tertiles, the mean levels of all three inflammatory markers generally increased.

### Exploratory analyses of menopause timing in relation to the inflammatory dietary score and biomarkers

Among the 1,426 postmenopausal women included in the menopause-related analyses, 147 had premature or early menopause (<45 years), and 1,279 had a normal menopausal age (≥45 years). As shown in Supplementary Table S9, compared with women of normal menopausal age, women with premature or early menopause had a higher standardized inflammatory dietary score [*β* = 0.229, 95% CI (0.085, 0.372), *p* = 0.002]. However, the relationship between the timing of menopause and inflammatory markers did not yield consistent results across the three indicators. Premature or early menopause was associated with higher log IL-6 levels [*β* = 0.137, 95% CI (0.002, 0.272), *p* = 0.046], but its associations with log CRP and log sTNFR-1 did not reach statistical significance (Supplementary Table S10).

Further interaction analysis showed that menopause timing did not significantly modify the relationship between the RRR-derived inflammatory dietary score and inflammatory biomarkers. As shown in Supplementary Table S11, the interaction term between the diet score and premature or early menopause did not reach statistical significance in the models for log CRP, log IL-6, and log sTNFR-1. Stratified analysis results showed that among women of normal menopausal age, a higher inflammatory dietary score was positively correlated with all three inflammatory biomarkers; among women with premature or early menopause, the inflammatory dietary score was positively correlated only with log IL-6, while the associations with log CRP and log sTNFR-1 were not statistically significant (Supplementary Table S12). Given that none of the interaction tests were significant, the stratified results should be considered primarily as descriptive supplements and should not be interpreted as indicating that menopause timing significantly alters the association between the inflammatory dietary score and the biomarkers.

### Food group contributions to the higher score among women with premature or early menopause

To further characterize the food sources contributing to higher RRR-oriented inflammatory dietary scores among women with premature or early menopause, this study compared the adjusted differences in score-contributing food groups across different menopause timing groups. As shown in Supplementary Table S13, women with premature or early menopause generally exhibited a trend of lower intake of some RRR-negative-loading food groups and higher intake of some RRR-positive-loading food groups. Specifically, intake of processed meat was significantly higher [*β* = 0.170, 95% CI (0.030, 0.310), *p* = 0.017], while intake of whole fruits was significantly lower [*β* = −0.147, 95% CI (−0.283, −0.012), *p* = 0.033]. In addition, intake levels for negative-loading food groups—such as nuts, fish and seafood, alcoholic beverages, cruciferous vegetables, leafy green vegetables, other vegetables, and whole grains—were generally lower, while intake levels for positive-loading food groups—such as sugar-sweetened beverages and refined grains—were higher; however, these differences were not statistically significant.

**Figure 2:**
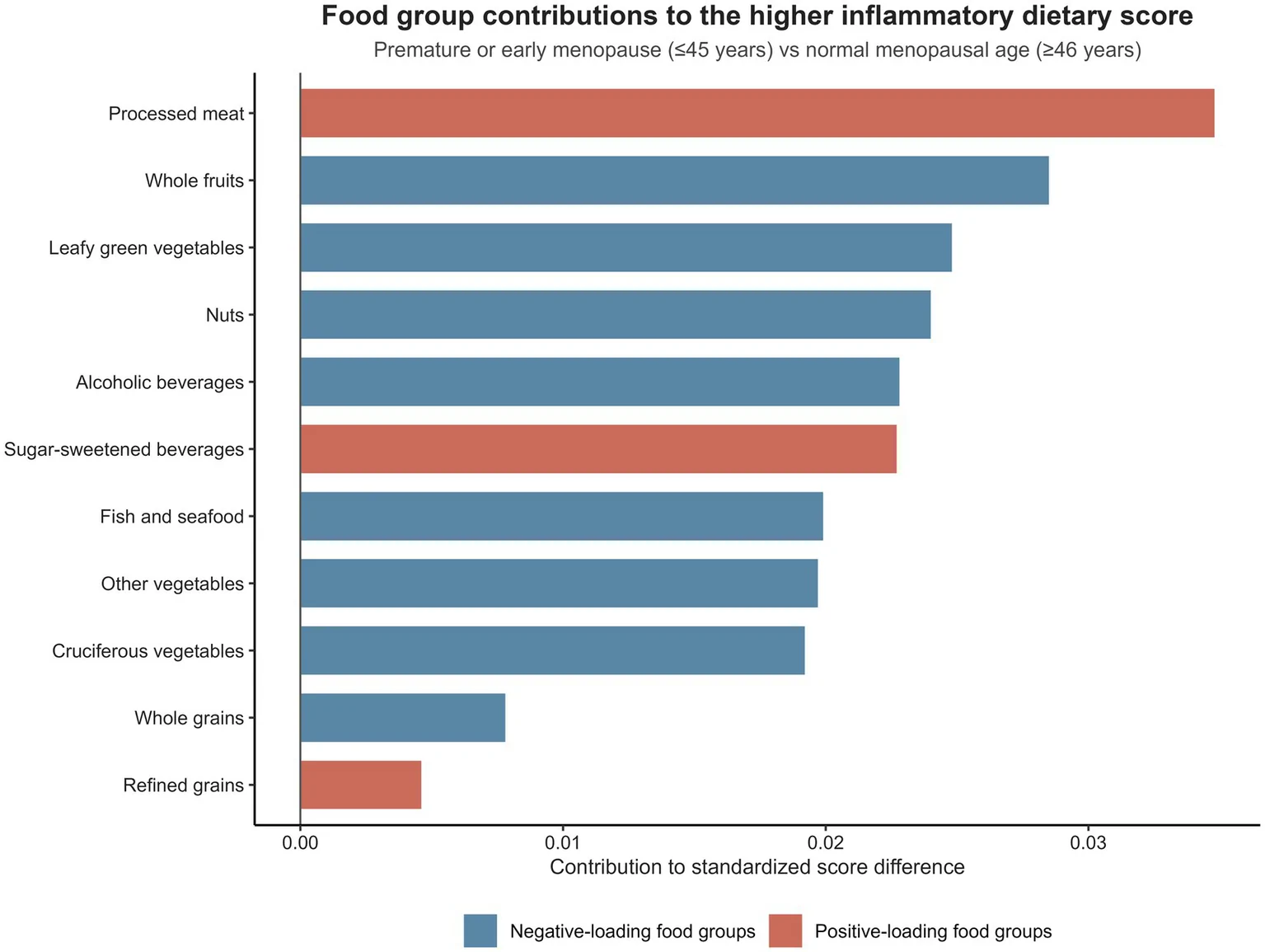
Bar chart illustrating food group contributions to higher inflammatory dietary scores comparing premature or early menopause to normal menopausal age. Processed meat and sugar-sweetened beverages are the major positive-loading contributors, while whole fruits, leafy green vegetables, nuts, and other listed foods are negative-loading groups. Legend identifies blue as negative-loading food groups and red as positive-loading food groups. X-axis represents contribution to standardized score difference; y-axis lists food group names. RRR, reduced rank regression.

Further contribution analyses revealed that the higher inflammatory dietary score observed in women with premature or early menopause resulted primarily from the combined contributions of differences across multiple food groups, rather than being entirely driven by a single food group. As shown in Supplementary Table S14 and Figure 2, the food groups with the largest relative contributions, in descending order, were processed meat (15.2%), whole fruits (12.5%), leafy green vegetables (10.8%), nuts (10.5%), alcoholic beverages (10.0%), and sugar-sweetened beverages (9.9%). Overall, this higher score primarily reflects higher intake of processed meat and lower intake of certain fruits and vegetables, accompanied by differences in the same direction across other score-contributing food groups. Since this analysis involves comparisons across multiple food groups and most differences within individual food groups were not statistically significant, the above results should be regarded as an exploratory decomposition of the sources of variation in the composite dietary score and should not be interpreted as indicating the independent effects of individual food groups.

## Discussion

Based on a sample of postmenopausal women from the HRS, this study constructed and characterized inflammation-oriented dietary patterns using a combined approach of PCA and RRR. PCA identified three natural dietary patterns: plant-rich, Western-style processed-snack, and animal-fat and condiment. Building on this, RRR further extracted inflammation-oriented dietary patterns associated with CRP, IL-6, and sTNFR-1, and developed standardized dietary scores based on these patterns. This RRR pattern is primarily characterized by higher intakes of processed meat, sugar-sweetened beverages, and refined grains, while intakes of nuts, fish and seafood, fruits, vegetables, and whole grains are lower. Comparison with PCA results revealed that the RRR pattern overlaps most with the plant-rich dietary pattern, but with opposite loadings; simultaneously, it exhibits partial co-directional overlap with the Western-style processed-snack dietary pattern, primarily reflected in food groups such as processed meat, refined grains, and sugar-sweetened beverages. Therefore, the inflammation-oriented dietary pattern identified in this study should not be simply equated with any specific natural dietary pattern; rather, it is better understood as a comprehensive dietary dimension related to inflammatory phenotypes, derived from the natural dietary structure of postmenopausal women.

These dietary patterns are generally consistent with previous research on dietary patterns and inflammatory markers. A study from the Nurses’ Health Study showed that a prudent dietary pattern was associated with lower levels of CRP and E-selectin, whereas a Western dietary pattern was associated with higher levels of CRP, IL-6, and various markers of endothelial function (22). In postmenopausal women, studies have also observed that dietary patterns characterized by higher intakes of fruits and vegetables are associated with lower levels of inflammation, whereas dietary patterns characterized by fast food consumption are associated with higher CRP levels (13). Recent systematic reviews and meta-analyses have also suggested that Western-style or meat-based dietary patterns are typically associated with higher levels of low-grade inflammation, whereas vegetable- and fruit-based, healthy, or Mediterranean dietary patterns are often associated with lower levels of inflammatory markers (23, 24). Furthermore, recent studies based on the HRS-HCNS and VBS datasets have linked overall dietary quality and plant-based dietary characteristics to health outcomes in older adults, such as composite biological risk and multimorbidity (15, 17). Taken together, this evidence supports understanding inflammation and chronic health risks from the perspective of overall dietary composition. Compared with previous studies, this study further incorporated CRP, IL-6, and sTNFR-1 as response variables into the RRR model extraction process, enabling the resulting dietary patterns to more directly reflect the inflammatory phenotype in our study sample. It should be noted that, since these inflammatory markers are also response variables in the RRR model, the association between dietary scores and inflammatory indicators should be understood as an internal characterization of inflammation-oriented dietary patterns rather than independent validation.

In this study, the RRR model’s positive-load food groups included processed meat, sugar-sweetened beverages, and refined grains. These food groups may collectively reflect higher levels of processing, higher intake of added sugars or refined carbohydrates, and exposure to certain processing- or cooking-related components. Previous studies have shown that consumption of sugar-sweetened beverages is associated with higher levels of CRP, IL-6, and tumor necrosis factor (TNF) receptor-related markers (25). As a complex processed food, processed meat may involve multiple exposures, such as salt curing, smoking, nitrite treatment, and heat-processing-related byproducts; these factors may contribute to health risks through pathways such as oxidative stress, inflammatory responses, or metabolic abnormalities (26–28). The positive load for refined grains should also be interpreted with caution. Compared to whole grains, refined grains typically contain lower levels of dietary fiber and certain micronutrients, and may reflect higher intake of refined carbohydrates or lower levels of whole grain substitution. Some studies have observed that higher refined grain intake is associated with higher CRP levels (29); however, differences across studies in grain definitions, control or substitute foods, co-consumption patterns, and overall dietary quality may influence the association between refined grains and inflammatory markers (29, 30). Therefore, these foods with positive loadings are better understood as common dietary characteristics within inflammation-promoting dietary patterns rather than being attributed to the independent pro-inflammatory effects of a single nutrient or food.

Conversely, foods with negative loadings in the RRR model include nuts, fish and seafood, fruits, vegetables, and whole grains. Lower intake of these foods may reflect poor overall dietary quality, rather than a deficiency in any single nutrient. A diet rich in plant-based foods, whole grains, nuts, and fish typically provides a variety of components, including dietary fiber, unsaturated fatty acids, vitamins, minerals, and phytochemicals. These components may be associated with lower systemic inflammation through mechanisms such as improving lipid and glucose metabolism, reducing oxidative stress, and regulating gut microbiota metabolism and inflammatory signaling (24, 31). However, evidence regarding individual foods or nutrients is not entirely consistent. For example, a meta-analysis of randomized trials examining the effects of nut intake on inflammatory markers such as CRP and IL-6 did not show consistent, significant improvements (32). Therefore, the findings of this study should emphasize “low intake of plant-based foods and high-quality food intake” as characteristics of an overall dietary pattern, rather than interpreting a single food group in isolation as a protective factor.

The overlap between PCA and RRR further illustrates the position of this inflammation-oriented pattern within natural dietary patterns. The RRR pattern showed the strongest overlap with the plant-rich dietary pattern but in the opposite direction, suggesting that higher inflammatory dietary scores may largely reflect a deviation from the plant-rich dietary pattern; at the same time, it exhibited a positive overlap with the Western-style processed-snack dietary pattern in food groups such as processed meat, refined grains, and sugar-sweetened beverages, indicating that this pattern also incorporates some characteristics of a typical processed diet. These findings support interpreting the RRR inflammation-oriented dietary pattern as a composite dimension jointly constituted by “low plant-based dietary characteristics” and “partial Western-style processed dietary characteristics,” rather than simply labeling it as the opposite of the Western dietary pattern or the plant-rich dietary pattern.

In the analysis specific to women, those with premature or early menopause had higher RRR inflammation-oriented dietary scores, accompanied by higher log IL-6 levels; however, consistent results were not observed for log CRP and log sTNFR-1, and menopause timing did not significantly modify the relationship between dietary scores and inflammatory markers. Therefore, findings related to menopause timing should be interpreted with caution. Since the timing of menopause was based on retrospective reports, and dietary intake likely reflected current dietary patterns measured many years after women entered menopause, these results are best interpreted as cross-sectional differences in current dietary patterns and certain inflammatory markers among women at different menopausal timings, rather than as a prospective effect of premature or early menopause on subsequent dietary behavior or inflammatory status. In other words, this study does not demonstrate that earlier menopause leads to higher levels of inflammation, nor does it demonstrate that it alters the relationship between dietary scores and inflammatory markers.

These findings may also be influenced by sociodemographic and lifestyle factors. Table 1 shows that the group with higher inflammation-promoting dietary scores had a higher proportion of women with lower educational attainment and Black/African American women, suggesting that this score is to some extent intertwined with sociodemographic characteristics. Previous studies have shown that factors such as educational level, smoking, employment status, and overall health status are associated with the age of natural menopause (33); simultaneously, factors such as education, income, living environment, and racial background are also associated with dietary quality (34). Therefore, the higher inflammation-promoting dietary scores observed among women with premature or early menopause may partially reflect residual confounding from socioeconomic, racial, and lifestyle factors. Even though this study adjusted for variables such as educational attainment, race, marital status, smoking, alcohol consumption, BMI, and chronic diseases, the influence of these factors cannot be completely ruled out. Consequently, the findings related to menopause timing are best regarded as exploratory supplementary data. Their primary significance lies in suggesting that women with premature or early menopause may constitute a subgroup worthy of attention in future dietary pattern assessments, rather than establishing a definitive causal relationship between the timing of menopause, dietary patterns, and inflammatory markers.

Further analysis of food group contributions revealed that the higher inflammation-promoting dietary scores observed in women with premature or early menopause were primarily driven by higher intake of processed meat and lower intake of whole fruits, accompanied by consistent differences in other score-contributing food groups. Since processed meat is a food with a positive loading and whole fruits are foods with a negative loading, differences in the intake of these two food groups between groups would both contribute to a higher inflammation-oriented dietary score. In light of the aforementioned sociodemographic and lifestyle context, these dietary differences may, to some extent, reflect current dietary patterns shaped by the combined influence of socioeconomic background, food accessibility, and lifestyle factors; however, they do not necessarily represent dietary characteristics unique to women with premature or early menopause. Previous studies have also suggested that food prices and dietary costs may influence dietary choices in the context of socioeconomic disparities; low-cost, high-energy-density foods are more likely to be chosen, while access to and sustained consumption of nutrient-dense foods such as fruits and vegetables may be constrained by economic and environmental factors (35, 36). Therefore, the higher intake of processed meat and lower intake of whole fruits observed in this study may be better understood as components of less favorable dietary patterns in the current dietary structure of women with earlier menopause, rather than as the independent effects of individual food groups on the timing of menopause or inflammation levels.

It should be noted that the food group contribution analysis remains an exploratory decomposition. Although the adjusted differences for processed meat and whole fruits reached statistical significance, this analysis involves comparisons across multiple food groups, and differences for most individual food groups did not reach statistical significance. Therefore, these results are better suited to illustrate the composition of differences in composite scores rather than being interpreted as independent effects of individual food groups. Furthermore, alcoholic beverages exhibited a negative loading in the RRR model of this study and accounted for a certain proportion in the contribution decomposition; this result should also be interpreted with caution. Previous studies suggest that alcohol consumption may be associated with socioeconomic background, overall lifestyle, and residual confounders (37, 38); therefore, this negative loading should be viewed as a component of the data-driven dietary pattern and should not be interpreted as indicating that alcohol consumption has anti-inflammatory effects or should be recommended.

This study has several strengths. First, based on a sample of postmenopausal women from the HRS, this study combines dietary data with multiple inflammatory biomarkers to examine the relationship between dietary patterns and inflammatory phenotypes in postmenopausal women at the population level. Second, this study employs a combination of two methods: PCA and RRR. PCA is used to describe the naturally emerging major dietary structures within the study population, while RRR further extracts inflammation-oriented dietary patterns associated with CRP, IL-6, and sTNFR-1, ensuring that the interpretation of dietary patterns incorporates both population-level structural context and inflammation-phenotype specificity. Furthermore, this study conducted an exploratory analysis of the relationship between menopause timing and inflammation-oriented dietary scores, as well as their association with inflammatory biomarkers. It also performed an exploratory breakdown of the food groups with higher scores among women with premature or early menopause, providing supplementary information for understanding inflammation-related dietary patterns in postmenopausal women.

This study also has several limitations. First, this study used dietary data from the 2013 HCNS and inflammatory marker data from the 2016 VBS, with a time lag of approximately three years between the two. Although this design allowed dietary assessment to be completed before the measurement of inflammatory markers, this time lag may also lead to exposure misclassification. For postmenopausal middle-aged and older women, new-onset diseases, hospitalizations, changes in body weight, changes in medication use, or lifestyle adjustments may occur within three years, all of which could alter their original dietary patterns. Therefore, the dietary patterns measured in 2013 may not fully represent their long-term dietary status around the time of the 2016 inflammatory marker testing. At the same time, health status or underlying inflammatory status during the study period may also have conversely influenced subsequent dietary behavior; therefore, the possibility of reverse causality or health-related dietary changes cannot be ruled out. For the reasons outlined above, the results of this study should be interpreted as an association between dietary patterns in 2013 and inflammatory marker levels in 2016, rather than as a causal effect of dietary patterns on subsequent inflammatory levels. Future studies should incorporate repeated dietary assessments and repeated measurements of inflammatory markers to more clearly evaluate the temporal relationship between changes in dietary composition and inflammatory trajectories.

Second, as an observational study, this study cannot completely rule out residual confounding. Dietary intake was assessed using a food frequency questionnaire, which may be subject to recall bias and measurement error; the categorization of food groups and standardization procedures may also affect the results of dietary pattern extraction. Furthermore, because the HCNS and VBS data were collected relatively early, the results of this study may not fully reflect recent changes in food availability, consumption of processed foods, and dietary behaviors among postmenopausal women. Dietary patterns extracted using RRR also depend on the study population, food group categorization, and the selected outcome variables; therefore, the inflammation-oriented dietary patterns established in this study require further validation in other samples of postmenopausal women, particularly in more recent external cohorts with repeated-measure data. Finally, age at menopause was primarily based on self-reports, which may be subject to recall bias; due to limitations in the variables available in the Harmonized HRS, this study was unable to reliably distinguish between bilateral oophorectomy and medically induced menopause, so some misclassification of menopause type may still exist. Stratification and interaction analyses related to premature or early menopause may also be limited by sample size. The food group contribution analysis was primarily used to explain possible sources of differences in the composite dietary score. Although between-group differences for “processed meat” and “whole fruits” reached statistical significance, this analysis cannot be used to infer the independent effects or causal relationships of individual food groups.

## Conclusion

This study developed an RRR-oriented inflammatory dietary score among postmenopausal women in the HRS. This score reflects a dietary profile characterized by high intake of processed meat, sugar-sweetened beverages, and refined grains, as well as low intake of nuts, fish and seafood, fruits, vegetables, and whole grains, and was positively associated with levels of CRP, IL-6, and sTNFR-1. A comparison of PCA results with the RRR model suggests that this pattern is primarily characterized by a deviation from a plant-rich dietary pattern and incorporates some features of a Western-style processed-snack dietary pattern. Exploratory analyses revealed that women who experienced premature or early menopause had higher inflammation-oriented dietary scores; however, the association between the timing of menopause and inflammatory markers was inconsistent, and no significant interaction was observed. These findings suggest that nutritional assessments of postmenopausal women should focus on comprehensive dietary characteristics, such as high intake of processed/refined foods and insufficient intake of plant-based foods, fish, and whole grains; women who experience early menopause may be a subgroup warranting special attention in future dietary pattern evaluations. Before this score can be translated into targeted dietary recommendations, it requires further longitudinal studies and validation in external populations.

## Data Availability

Publicly available datasets were analyzed in this study. This data can be found at: https://hrs.isr.umich.edu/.
