# Supplementary material for: Inflammation-oriented dietary pattern and menopause timing in postmenopausal women: evidence from the Health and Retirement Study
Source: Front Nutr. 2026 Aug 12;13:1905625. doi: 10.3389/fnut.2026.1905625 (PMC13506304; doi:10.3389/fnut.2026.1905625)
Supplement: Supplementary file 1 [file Table_1.DOCX]

**Supplementary Materials**

**Inflammation-oriented dietary pattern and menopause timing in postmenopausal women: evidence from the Health and Retirement Study**

Supplementary Table S1. PCA adequacy and component retention criteria

Supplementary Table S2. Comparison of two- to five-component PCA solutions

Supplementary Table S3. Variance explained by the final three-component PCA solution

Supplementary Table S4. Varimax-rotated loading matrix for the final three-component PCA solution

Supplementary Table S5. Loadings of the RRR-derived inflammatory dietary pattern

Supplementary Table S6. Overlap between the RRR-derived inflammatory dietary pattern and PCA-derived dietary patterns

Supplementary Table S7. Food groups overlapping between PCA-derived dietary patterns and the RRR-derived inflammatory pattern

Supplementary Table S8. Pearson correlations between the RRR-derived inflammatory dietary score and inflammatory biomarkers

Supplementary Table S9. Association between premature or early menopause and the RRR-derived inflammatory dietary score

Supplementary Table S10. Association between premature or early menopause and inflammatory biomarkers

Supplementary Table S11. Effect modification by menopause timing in the associations between the RRR-derived inflammatory dietary score and inflammatory biomarkers

Supplementary Table S12. Stratified associations of the RRR-derived inflammatory dietary score with inflammatory biomarkers by menopause timing

Supplementary Table S13. Adjusted differences in score-contributing food groups by menopause timing

Supplementary Table S14. Food group contributions to the difference in the RRR-derived inflammatory dietary score by menopause timing

Supplementary Figure S1. Flowchart of participant selection

Supplementary Figure S2. Scree plot for principal component analysis of food groups

Supplementary Figure S3. Overlap between PCA-derived dietary patterns and the RRR-derived inflammatory dietary pattern

Supplementary Figure S4. Inflammatory biomarker levels across tertiles of the RRR-derived inflammatory dietary score

**Supplementary Table S1. PCA adequacy and component retention criteria**

| **Measure** | **Value** |
| --- | --- |
| Kaiser-Meyer-Olkin measure | 0.9 |
| Bartlett test chi-square | 13556.26 |
| Bartlett test df | 780 |
| Bartlett test p value | < 0.001 |
| Number of components with eigenvalue > 1.25 | 5 |
| Final retained solution | 3-factor solution |

Note: Abbreviations: KMO, Kaiser-Meyer-Olkin measure; PCA, principal component analysis.

**Supplementary Table S2. Comparison of two- to five-component PCA solutions**

| **Solution** | **Number of factors** | **Total SS loadings** | **Total variance explained, %** | **Minimum number of salient food groups per factor** | **Maximum number of salient food groups per factor** |
| --- | --- | --- | --- | --- | --- |
| 2-factor | 2 | 10.408 | 26.02 | 21 | 23 |
| 3-factor | 3 | 12.070 | 30.17 | 16 | 22 |
| 4-factor | 4 | 13.540 | 33.85 | 11 | 20 |
| 5-factor | 5 | 14.948 | 37.37 | 9 | 18 |

Note: Salient food groups were defined as those with absolute varimax-rotated loadings ≥0.25. Although the four- and five-factor solutions explained a higher proportion of variance, the three-factor solution was retained because it provided a more parsimonious and nutritionally interpretable structure, with sufficient salient food groups in each factor. Abbreviations: SS, sum of squares. PCA, principal component analysis.
 **Supplementary Table S3. Variance explained by the final three-component PCA solution**

| **Factor** | **SS loadings** | **Variance explained, %** | **Cumulative variance, %** |
| --- | --- | --- | --- |
| Plant-rich dietary pattern | 5.226 | 13.07 | 13.07 |
| Western-style processed-snack dietary pattern | 4.091 | 10.23 | 23.29 |
| Animal-fat and condiment dietary pattern | 2.753 | 6.88 | 30.17 |

Note: Variance explained refers to the proportion of total food group variance explained by each varimax-rotated component. The cumulative variance explained by the three retained patterns was 30.17%, indicating that these components summarized the major dietary structures in the study sample but did not capture all individual variability in food group intake. Abbreviations: SS, sum of squares. PCA, principal component analysis.

**Supplementary Table S4. Varimax-rotated loading matrix for the final three-component PCA solution**

| **Food group** | **Plant-rich dietary pattern** | **Western-style processed-snack dietary pattern** | **Animal-fat and condiment dietary pattern** |
| --- | --- | --- | --- |
| Butter, margarine, and spreads |  |  | 0.530 |
| Red meat |  | 0.419 | 0.507 |
| Condiments and sauces | 0.259 |  | 0.440 |
| Eggs | 0.323 |  | 0.424 |
| Dressings and spreads |  |  | 0.423 |
| High-fat milk and cream |  |  | 0.341 |
| Cheese |  |  | 0.307 |
| Organ meat |  |  | 0.256 |
| Other vegetables | 0.756 |  | 0.297 |
| Whole fruits | 0.709 |  |  |
| Leafy green vegetables | 0.701 |  |  |
| Cruciferous vegetables | 0.652 |  |  |
| Legumes and soy products | 0.568 |  |  |
| Tomato products | 0.560 |  | 0.340 |
| Nuts | 0.532 |  |  |
| Fish and seafood | 0.475 | 0.293 |  |
| Fruit juice | 0.451 |  |  |
| Snack bars | 0.418 | 0.399 | -0.258 |
| Whole grains | 0.411 | 0.326 |  |
| Breakfast cereal and oatmeal | 0.402 |  |  |
| Alcoholic beverages | 0.397 | 0.313 |  |
| Seasonings | 0.368 |  | 0.308 |
| Yogurt | 0.351 |  |  |
| Poultry | 0.350 |  | 0.267 |
| Tea and coffee | 0.340 |  |  |
| Water | 0.319 |  |  |
| Healthy added fats | 0.316 |  |  |
| Mixed starches and fast foods |  | 0.629 |  |
| Salty snacks | 0.282 | 0.625 |  |
| Cookies, cakes, and pastries |  | 0.612 |  |
| Processed meat |  | 0.569 | 0.361 |
| Chocolate and candy |  | 0.568 |  |
| Potatoes, fries, and chips |  | 0.551 | 0.415 |
| Refined grains |  | 0.506 | 0.427 |
| Sugar-sweetened beverages |  | 0.439 |  |
| Cream soup |  | 0.403 |  |
| Ice cream and dairy desserts |  | 0.310 |  |
| Sweet spreads |  | 0.304 |  |
| Diet beverages |  | 0.273 |  |

Note: Only absolute factor loadings ≥0.25 are shown. Abbreviation: PCA, principal component analysis.

**Supplementary Table S5. Loadings of the RRR-derived inflammatory dietary pattern**

| **Food group** | **RRR loading** | **Absolute RRR loading** |
| --- | --- | --- |
| Nuts | -0.383 | 0.383 |
| Fish and seafood | -0.364 | 0.364 |
| Alcoholic beverages | -0.315 | 0.315 |
| Processed meat | 0.311 | 0.311 |
| Cruciferous vegetables | -0.306 | 0.306 |
| Whole fruits | -0.295 | 0.295 |
| Leafy green vegetables | -0.289 | 0.289 |
| Sugar-sweetened beverages | 0.282 | 0.282 |
| Other vegetables | -0.275 | 0.275 |
| Whole grains | -0.260 | 0.260 |
| Refined grains | 0.254 | 0.254 |

Note: Food groups with absolute RRR loadings ≥0.25 in the first RRR-derived pattern are shown. Positive loadings indicate food groups contributing to a higher inflammatory dietary score when intake is higher, whereas negative loadings indicate food groups contributing to a lower score when intake is higher. Abbreviations: CRP, C-reactive protein; IL-6, interleukin-6; RRR, reduced rank regression; sTNFR-1, soluble tumor necrosis factor receptor 1.

**Supplementary Table S6. Overlap between the RRR-derived inflammatory dietary pattern and PCA-derived dietary patterns**

| **PCA pattern** | **Number of overlapping food groups** | **Weighted absolute overlap** | **Signed alignment** | **Same direction, n** | **Opposite direction, n** | **Overlapping food groups** |
| --- | --- | --- | --- | --- | --- | --- |
| Plant-rich dietary pattern | 8 | 1.428 | -1.428 | 0 | 8 | Whole fruits; Other vegetables; Nuts; Leafy green vegetables; Cruciferous vegetables; Fish and seafood; Alcoholic beverages; Whole grains |
| Western-style processed-snack dietary pattern | 6 | 0.719 | 0.139 | 3 | 3 | Processed meat; Refined grains; Sugar-sweetened beverages; Fish and seafood; Alcoholic beverages; Whole grains |
| Animal-fat and condiment dietary pattern | 3 | 0.303 | 0.139 | 2 | 1 | Processed meat; Refined grains; Other vegetables |

Note: Overlapping food groups were identified using the prespecified loading thresholds in both PCA and RRR analyses. Weighted absolute overlap reflects the overall magnitude of overlap, while signed alignment retains the direction of the PCA and RRR loadings. Abbreviations: PCA, principal component analysis; RRR, reduced rank regression.

**Supplementary Table S7. Food groups overlapping between PCA-derived dietary patterns and the RRR-derived inflammatory pattern**

| **PCA pattern** | **Food group** | **PCA loading** | **RRR loading** | **Direction relation** | **Weighted overlap** |
| --- | --- | --- | --- | --- | --- |
| Animal-fat and condiment dietary pattern | Processed meat | 0.361 | 0.311 | Same direction | 0.113 |
| Animal-fat and condiment dietary pattern | Refined grains | 0.427 | 0.254 | Same direction | 0.108 |
| Animal-fat and condiment dietary pattern | Other vegetables | 0.297 | -0.275 | Opposite direction | 0.082 |
| Plant-rich dietary pattern | Whole fruits | 0.709 | -0.295 | Opposite direction | 0.209 |
| Plant-rich dietary pattern | Other vegetables | 0.756 | -0.275 | Opposite direction | 0.208 |
| Plant-rich dietary pattern | Nuts | 0.532 | -0.383 | Opposite direction | 0.204 |
| Plant-rich dietary pattern | Leafy green vegetables | 0.701 | -0.289 | Opposite direction | 0.203 |
| Plant-rich dietary pattern | Cruciferous vegetables | 0.652 | -0.306 | Opposite direction | 0.200 |
| Plant-rich dietary pattern | Fish and seafood | 0.475 | -0.364 | Opposite direction | 0.173 |
| Plant-rich dietary pattern | Alcoholic beverages | 0.397 | -0.315 | Opposite direction | 0.125 |
| Plant-rich dietary pattern | Whole grains | 0.411 | -0.260 | Opposite direction | 0.107 |
| Western-style processed-snack dietary pattern | Processed meat | 0.569 | 0.311 | Same direction | 0.177 |
| Western-style processed-snack dietary pattern | Refined grains | 0.506 | 0.254 | Same direction | 0.129 |
| Western-style processed-snack dietary pattern | Sugar-sweetened beverages | 0.439 | 0.282 | Same direction | 0.124 |
| Western-style processed-snack dietary pattern | Fish and seafood | 0.293 | -0.364 | Opposite direction | 0.107 |
| Western-style processed-snack dietary pattern | Alcoholic beverages | 0.313 | -0.315 | Opposite direction | 0.099 |
| Western-style processed-snack dietary pattern | Whole grains | 0.326 | -0.260 | Opposite direction | 0.085 |

Note: Weighted overlap was calculated as the product of the absolute PCA loading and the absolute RRR loading for each overlapping food group. Direction relation indicates whether the PCA and RRR loadings were in the same or opposite direction. Abbreviations: PCA, principal component analysis; RRR, reduced rank regression.

**Supplementary Table S8. Pearson correlations between the RRR-derived inflammatory dietary score and inflammatory biomarkers**

| **Variable** | **RRR-derived inflammatory dietary score** | **log CRP** | **log IL-6** | **log sTNFR-1** |
| --- | --- | --- | --- | --- |
| RRR-derived inflammatory dietary score | 1.000 | 0.112 | 0.148 | 0.180 |
| log CRP | 0.112 | 1.000 | 0.446 | 0.319 |
| log IL-6 | 0.148 | 0.446 | 1.000 | 0.388 |
| log sTNFR-1 | 0.180 | 0.319 | 0.388 | 1.000 |

Note: Values are Pearson correlation coefficients. Abbreviations: CRP, C-reactive protein; IL-6, interleukin-6; RRR, reduced rank regression; sTNFR-1, soluble tumor necrosis factor receptor 1.

**Supplementary Table S9. Association between premature or early menopause and the RRR-derived inflammatory dietary score**

| **Comparison** | **β (95% CI)** | ***P* value** |
| --- | --- | --- |
| Premature or early menopause vs normal menopausal age | 0.229 (0.085, 0.372) | 0.002 |

Note: Analyses included 147 women with premature or early menopause (<45 years) and 1,279 women with normal menopausal age (≥45 years). Normal menopausal age was used as the reference group. β represents the adjusted difference in the standardized RRR-derived inflammatory dietary score. The model was adjusted for age, total energy intake, education level, race, marital status, current smoking, current drinking, hypertension, diabetes, cancer, cardiovascular disease, and BMI category. Abbreviations: CI, confidence interval; RRR, reduced rank regression.

**Supplementary Table S10. Association between premature or early menopause and inflammatory biomarkers**

| **Outcome** | **Comparison** | **β (95% CI)** | ***P* value** |
| --- | --- | --- | --- |
| log CRP | Premature or early menopause vs normal menopausal age | 0.091 (-0.070, 0.253) | 0.268 |
| log IL-6 | Premature or early menopause vs normal menopausal age | 0.137 (0.002, 0.272) | 0.046 |
| log sTNFR-1 | Premature or early menopause vs normal menopausal age | 0.017 (-0.042, 0.076) | 0.576 |

Note: Analyses included 147 women with premature or early menopause (<45 years) and 1,279 women with normal menopausal age (≥45 years). β coefficients represent adjusted differences in log-transformed inflammatory biomarkers. Models were adjusted for age, total energy intake, education level, race, marital status, current smoking, current drinking, hypertension, diabetes, cancer, cardiovascular disease, and BMI category. Abbreviations: CI, confidence interval; CRP, C-reactive protein; IL-6, interleukin-6; sTNFR-1, soluble tumor necrosis factor receptor 1.

**Supplementary Table S11. Effect modification by menopause timing in the associations between the RRR-derived inflammatory dietary score and inflammatory biomarkers**

| **Outcome** | **Interaction** | **Interaction β (95% CI)** | ***P* for interaction** |
| --- | --- | --- | --- |
| log CRP | Diet score × premature or early menopause | -0.037 (-0.206, 0.133) | 0.672 |
| log IL-6 | Diet score × premature or early menopause | 0.050 (-0.090, 0.190) | 0.485 |
| log sTNFR-1 | Diet score × premature or early menopause | -0.013 (-0.074, 0.049) | 0.689 |

Note: Analyses included 147 women with premature or early menopause (<45 years) and 1,279 women with normal menopausal age (≥45 years). β coefficients represent interaction effects on log-transformed biomarker levels. Models included the RRR-derived inflammatory dietary score, menopause timing, and their interaction term, and were adjusted for age, total energy intake, education level, race, marital status, current smoking, current drinking, hypertension, diabetes, cancer, cardiovascular disease, and BMI category. Abbreviations: CI, confidence interval; CRP, C-reactive protein; IL-6, interleukin-6; RRR, reduced rank regression; sTNFR-1, soluble tumor necrosis factor receptor 1.

**Supplementary Table S12. Stratified associations of the RRR-derived inflammatory dietary score with inflammatory biomarkers by menopause timing**

| **Outcome** | **Menopause timing** | **β (95% CI)** | ***P* value** |
| --- | --- | --- | --- |
| log CRP | Normal menopausal age (≥45 years) | 0.085 (0.022, 0.148) | 0.008 |
| log CRP | Premature or early menopause (<45 years) | -0.008 (-0.213, 0.197) | 0.942 |
| log IL-6 | Normal menopausal age (≥45 years) | 0.119 (0.066, 0.171) | <0.001 |
| log IL-6 | Premature or early menopause (<45 years) | 0.202 (0.033, 0.370) | 0.020 |
| log sTNFR-1 | Normal menopausal age (≥45 years) | 0.044 (0.021, 0.068) | <0.001 |
| log sTNFR-1 | Premature or early menopause (<45 years) | 0.027 (-0.037, 0.091) | 0.406 |

Note: Analyses included 147 women with premature or early menopause (<45 years) and 1,279 women with normal menopausal age (≥45 years). β coefficients represent associations between each 1-standard-deviation higher RRR-derived inflammatory dietary score and log-transformed inflammatory biomarkers within each menopause timing group. Models were adjusted for age, total energy intake, education level, race, marital status, current smoking, current drinking, hypertension, diabetes, cancer, cardiovascular disease, and BMI category. Abbreviations: CI, confidence interval; CRP, C-reactive protein; IL-6, interleukin-6; RRR, reduced rank regression; sTNFR-1, soluble tumor necrosis factor receptor 1.

**Supplementary Table S13. Adjusted differences in score-contributing food groups by menopause timing**

| **Food group** | **RRR loading** | **RRR loading direction** | **Food intake direction in earlier menopause** | **Standardized difference (95% CI)** | **Interpretation for score** | ***P* value** |
| --- | --- | --- | --- | --- | --- | --- |
| Nuts | -0.383 | Negative loading | Lower intake | -0.095 (-0.251, 0.061) | Lower intake contributes to higher score | 0.232 |
| Fish and seafood | -0.364 | Negative loading | Lower intake | -0.083 (-0.231, 0.064) | Lower intake contributes to higher score | 0.268 |
| Alcoholic beverages | -0.315 | Negative loading | Lower intake | -0.110 (-0.254, 0.034) | Lower intake contributes to higher score | 0.134 |
| Processed meat | 0.311 | Positive loading | Higher intake | 0.170 (0.030, 0.310) | Higher intake contributes to higher score | 0.017 |
| Cruciferous vegetables | -0.306 | Negative loading | Lower intake | -0.095 (-0.253, 0.062) | Lower intake contributes to higher score | 0.233 |
| Whole fruits | -0.295 | Negative loading | Lower intake | -0.147 (-0.283, -0.012) | Lower intake contributes to higher score | 0.033 |
| Leafy green vegetables | -0.289 | Negative loading | Lower intake | -0.130 (-0.281, 0.020) | Lower intake contributes to higher score | 0.089 |
| Sugar-sweetened beverages | 0.282 | Positive loading | Higher intake | 0.123 (-0.032, 0.277) | Higher intake contributes to higher score | 0.121 |
| Other vegetables | -0.275 | Negative loading | Lower intake | -0.109 (-0.245, 0.027) | Lower intake contributes to higher score | 0.117 |
| Whole grains | -0.260 | Negative loading | Lower intake | -0.045 (-0.199, 0.108) | Lower intake contributes to higher score | 0.561 |
| Refined grains | 0.254 | Positive loading | Higher intake | 0.028 (-0.102, 0.158) | Higher intake contributes to higher score | 0.676 |

Note: Standardized differences compare food group intake between 147 women with premature or early menopause (<45 years) and 1,279 women with normal menopausal age (≥45 years). Standardized differences were estimated using multivariable linear regression adjusted for age, total energy intake, education level, race, marital status, current smoking, current drinking, hypertension, diabetes, cancer, cardiovascular disease, and BMI category. Positive values indicate higher standardized intake in women with premature or early menopause, whereas negative values indicate lower standardized intake. Abbreviations: CI, confidence interval; RRR, reduced rank regression.

**Supplementary Table S14. Food group contributions to the difference in the RRR-derived inflammatory dietary score by menopause timing**

| **Food group** | **Food group difference β** | **RRR loading** | **Score mechanism** | **Standardized contribution to score difference** | **Relative absolute contribution, %** | **Contribution direction** | ***P* value for food group difference** |
| --- | --- | --- | --- | --- | --- | --- | --- |
| Processed meat | 0.170 | 0.311 | Higher intake of positive-loading food | 0.0348 | 15.2 | Contributes to higher score in earlier menopause | 0.017 |
| Whole fruits | -0.147 | -0.295 | Lower intake of negative-loading food | 0.0285 | 12.5 | Contributes to higher score in earlier menopause | 0.033 |
| Leafy green vegetables | -0.130 | -0.289 | Lower intake of negative-loading food | 0.0248 | 10.8 | Contributes to higher score in earlier menopause | 0.089 |
| Nuts | -0.095 | -0.383 | Lower intake of negative-loading food | 0.0240 | 10.5 | Contributes to higher score in earlier menopause | 0.232 |
| Alcoholic beverages | -0.110 | -0.315 | Lower intake of negative-loading food | 0.0228 | 10.0 | Contributes to higher score in earlier menopause | 0.134 |
| Sugar-sweetened beverages | 0.123 | 0.282 | Higher intake of positive-loading food | 0.0227 | 9.9 | Contributes to higher score in earlier menopause | 0.121 |
| Fish and seafood | -0.083 | -0.364 | Lower intake of negative-loading food | 0.0199 | 8.7 | Contributes to higher score in earlier menopause | 0.268 |
| Other vegetables | -0.109 | -0.275 | Lower intake of negative-loading food | 0.0197 | 8.6 | Contributes to higher score in earlier menopause | 0.117 |
| Cruciferous vegetables | -0.095 | -0.306 | Lower intake of negative-loading food | 0.0192 | 8.4 | Contributes to higher score in earlier menopause | 0.233 |
| Whole grains | -0.045 | -0.260 | Lower intake of negative-loading food | 0.0078 | 3.4 | Contributes to higher score in earlier menopause | 0.561 |
| Refined grains | 0.028 | 0.254 | Higher intake of positive-loading food | 0.0046 | 2.0 | Contributes to higher score in earlier menopause | 0.676 |

Note: Contributions represent the estimated contribution of each food group to the standardized dietary score difference between 147 women with premature or early menopause (<45 years) and 1,279 women with normal menopausal age (≥45 years). Positive contributions indicate food groups contributing to the higher score among women with premature or early menopause. Relative absolute contribution indicates the proportion of the total absolute food group contribution attributable to each food group. P values refer to adjusted food group differences rather than to the contribution estimates. These results should be interpreted as an exploratory decomposition of the composite dietary score difference. Abbreviations: RRR, reduced rank regression.

**Supplementary Figure S1. Flowchart of participant selection**

Note: Abbreviation: CRP, C-reactive protein; HCNS, Health Care and Nutrition Study; HRS, Health and Retirement Study; IL-6, interleukin-6; sTNFR-1, soluble tumor necrosis factor receptor 1.

**Supplementary Figure S2. Scree plot for principal component analysis of food groups**

Note: The dashed horizontal line indicates the eigenvalue threshold of 1.25, and the vertical line indicates the final retained three-component solution.

**Supplementary Figure S3. Overlap between PCA-derived dietary patterns and the RRR-derived inflammatory dietary pattern**

Note: Dot size represents the weighted overlap between PCA and RRR loadings for each food group, and dot color represents the direction and magnitude of the RRR loading. Abbreviations: PCA, principal component analysis; RRR, reduced rank regression.

**Supplementary Figure S4. Inflammatory biomarker levels across tertiles of the RRR-derived inflammatory dietary score**

Note: Points represent mean log-transformed biomarker levels within each dietary score tertile, and error bars represent 95% confidence intervals. Abbreviations: CI, confidence interval; CRP, C-reactive protein; IL-6, interleukin-6; RRR, reduced rank regression; sTNFR-1, soluble tumor necrosis factor receptor 1.
